# New CagL Amino Acid Polymorphism Patterns of *Helicobacter pylori* in Peptic Ulcer and Non-Ulcer Dyspepsia

**DOI:** 10.3390/medicina58121738

**Published:** 2022-11-27

**Authors:** Reyhan Caliskan, Silva Polat Sari, Bahadir Ercan, Kivanc Derya Peker, Mehtap Omac Sonmez, Ozer Akgul, Burcu Sapmaz, Aliye Soylu, Gokhan Tolga Adas, Yasar Ali Oner, Pelin Yuksel Mayda

**Affiliations:** 1 Department of Medical Microbiology, School of Medicine, Istanbul Aydin University, Istanbul 34295, Turkey; 2 Vocational School of Health Services, Istanbul Aydin University, Istanbul 34295, Turkey; 3 Department of Biochemistry, School of Medicine, Istanbul Aydin University, Istanbul 34295, Turkey; 4 Department of General Surgery, Hisar Intercontinental Hospital, Istanbul 34768, Turkey; 5 Faculty of Health Science, Kahramanmaras Sutcu Imam University, Kahramanmaras 46050, Turkey; 6 Department of Gastroenterology, Bagcilar Medipol Mega University Hospital, Istanbul Medipol University, Istanbul 34810, Turkey; 7 Department of Surgery, Bakirkoy Dr. Sadi Konuk Training and Research Hospital, University of Health Sciences, Istanbul 34147, Turkey; 8 Faculty of Pharmacy, Kocaeli Health and Technology University, Basiskele 41275, Turkey

**Keywords:** *Helicobacter pylori*, CagL polymorphisms, CagLHM, peptic ulcer, non-ulcer dyspepsia

## Abstract

*Background and Objectives*: *Helicobacter pylori* infection is associated with chronic gastritis, ulcers, and gastric cancer. The *H. pylori* Type 4 secretion system (T4SS) translocates the CagA protein into host cells and plays an essential role in initiating gastric carcinogenesis. The CagL protein is a component of the T4SS. CagL amino acid polymorphisms are correlated with clinical outcomes. We aimed to study the association between CagL amino acid polymorphisms and peptic ulcer disease (PUD) and non-ulcer dyspepsia (NUD). *Materials and Methods*: A total of 99 patients (PUD, 46; NUD, 53) were enrolled and screened for *H. pylori* by qPCR from antrum biopsy samples. The amino acid polymorphisms of CagL were analyzed using DNA sequencing, followed by the MAFFT sequence alignment program to match the amino acid sequences. *Results*: Antrum biopsy samples from 70 out of 99 (70.7%) patients were found to be *H. pylori* DNA-positive. A positive band for *cagL* was detected in 42 out of 70 samples (PUD, 23; NUD, 19), and following this, these 42 samples were sequenced. In total, 27 different polymorphisms were determined. We determined three CagL amino acid polymorphism combinations, which were determined to be associated with PUD and NUD. Pattern 1 (K35/N122/V134/T175/R194/E210) was only detected in PUD patient samples and was related to a 1.35-fold risk (*p* = 0.02). Patterns 2 (V41/I134) and 3 (V41/K122/A171/I174) were found only in NUD patient samples and were linked to a 1.26-fold increased risk (*p* = 0.03). *Conclusions*: We observed three new patterns associated with PUD and NUD. Pattern 1 is related to PUD, and the other two patterns (Patterns 2 and 3) are related to NUD. The patterns that we identified include the remote polymorphisms of the CagL protein, which is a new approach. These patterns may help to understand the course of *H. pylori* infection.

## 1. Introduction

*Helicobacter pylori* colonizes the gastric mucosa, causing asymptomatic colonization, chronic gastritis, ulcers, or gastric cancer [1]. While many bacteria are only considered infectious agents, *H. pylori* is well known for causing gastric cancer. The worldwide prevalence of *H. pylori* infection is important not only for gastric cancer but also for the economic burden of treatment. Although many virulence factors are considered to play a role in *H. pylori*’s pathogenesis, CagA protein and the EPIYA motif coded in *cag* pathogenicity islands (*cag*PAI) have been proven to be associated with gastric cancer [2,3,4,5,6]. The colonization of *H. pylori* is the first step in its pathogenicity, but the events that happen thereafter seem more crucial. Briefly, after passing the mucin barrier, *H. pylori* colonizes close to the epithelial layer and mostly adheres to epithelial cells. Via the Type 4 secretion system (T4SS), CagA proteins are transferred into epithelial cells [2,7]. In this transfer process, it has been shown that the CagL protein, a T4SS protein, is crucial [8]. After being transferred to the cell, the phosphorylation of the CagA protein on tyrosine residues is associated with the spreading of the cells and thus the development of gastric cancer [3,9,10,11].

If we focus on *H. pylori* infection, which starts with the adherence of bacteria to the epithelial cell surface, there are some key events and proteins. CagL protein has been investigated extensively, and the role of different amino acid polymorphisms has been assessed in many studies. One of the motifs on CagL is RGD (arginine, glycine, and aspartate; 76–78th residues on the protein), which interacts with the integrins α5β1, α5β5, and α5β3 on the epithelial cell [12,13,14]. These interactions result in IL-8 secretion, which triggers cell spreading, focal adhesion, and the activation of several tyrosine kinases [15,16]. There is an RGD helper sequence (RHS) that is composed of phenylalanine–glutamic acid–alanine–asparagine–glutamic acid (FEANE 86–90) that helps the RGD sequence bind integrin α5β1 [17]. L79 and L82 (two leucine residues) form another functional motif called LXXL, which allows bacteria to adhere to cell lines via integrin α5β6 [18]. Another motif called TASLI (threonine–alanine–serine–leucine–isoleucine 170–174) binds to integrins in an RGD-independent manner. Its deletion is related to reduced CagL binding to integrins, leading to reduced IL-8 secretion and CagA translocation [8]. Aside from these important motifs, there is a highly variable region on positions 58–62 called the CagL hypervariable motif (CagLHM). It has been shown that polymorphisms in this region affect the binding affinity of CagL to integrin α5β1 [19]. Polymorphisms in this region are quite important, and they are mostly associated with gastroduodenal diseases such as gastric cancer, chronic gastritis, and peptic ulcer [20,21,22,23,24,25,26]. Additionally, CagLHM amino acid polymorphisms show a geographic distribution similar to EPIYA-C/D [27]. Gorrell et al. [27] determined 33 motifs, of which DKMGE, NEIGQ, NKIGQ, and DKIGK are present in 75% of all strains worldwide. DKMGE is especially prevalent in Africa and America, but not in Asia. Other motifs, such as NEIGQ and NKIGQ, are dominant in Europe. CagLHM shows great variability in Asia, and the NEIGQ motif is prevalent in Western Asia, whereas DKIGK is dominant in Eastern Asia. Due to the diversity in this region, IL-8 response also varies between Eastern and Western Asia, as shown by Choi et al. [28].

Although there have been many studies focused on CagL amino acid polymorphisms related to gastroduodenal diseases, it is obvious that many more studies are needed. Although *H. pylori* infection is widely spread in our region and has been studied well in areas such as epidemiology, antibiotic resistance, and immunopathogenesis, data on CagL amino acid polymorphisms are limited. Due to the lack of data, we aimed to study the association between CagL amino acid polymorphisms and peptic ulcer disease (PUD) and non-ulcer dyspepsia (NUD).

## 2. Materials and Methods

### 2.1. Study Design

This study was planned as a cross-sectional study. Study subjects were selected among patients with dyspepsia symptoms who applied to the endoscopy unit of the gastroenterology clinics at Istanbul Bakirkoy Dr. Sadi Konuk Training and Research Hospital. A total of 99 patients (PUD, 46; NUD, 53) who had been diagnosed by a gastroenterologist via endoscopic evaluation were enrolled in the study. Antrum and corpus biopsy samples were taken from patients between April 2019 and February 2020.

Patients younger than 18 years old who had undergone gastric surgery, received *H. pylori* eradication treatment, consumed antibiotics in the previous month, consumed anti-secretory drugs, bismuth salts, or sucralfate in the last 2 weeks, or had a history of bleeding and/or coagulation disorders were excluded from this study.

All the patients signed an informed consent form, and the study was approved by the Istanbul Aydin University Ethics Committee (2019/82).

### 2.2. DNA Extraction

In this study, 200 mg of antrum biopsy samples were used for genomic DNA extractions. First, samples were transferred into a lysis solution (0.5 μg/μL Proteinase K, 5% Tween 20, 3 M guanidinium thiocyanate, 20 mM Tris-HCl, pH 8.0) and incubated at 70 °C for 15 min, followed by additional incubation at 95 °C for 5 min. Then, 500 µL of isopropanol was added before being transferred into a silica column and centrifuged at maximum speed for 2 min. DNA columns were washed twice with a washing solution (20 mM NaCl, 2 mM Tris-HCl 80% ethanol, pH 7.5). Then, DNA samples were eluted in 50 μL of elution solution (100 mM Tris-HCl, pH 8.0) and stored at −20 °C until further analysis.

### 2.3. H. pylori Detection

We used qPCR to detect *H. pylori* DNA in the samples; forward and reverse primers (5′-GCTCTCACTTCCATAGGCTATAATGTG-3′ and 5′-GCGCATGTCTTCGGTTAAAAA-3′, respectively) designed by Saez et al. were used to detect the urease gene [29]. qPCR reactions were performed according to the manufacturer’s recommendations (Premix Ex Taq Mastermix, Takara Bio Inc., Shiga, Japan) using a BioRad CFX96 Real-Time PCR Detection System (Bio-Rad Laboratories, Hercules, California, USA). The amplification conditions were as follows: initial denaturation at 95 °C for 30 s, 40 cycles of 95 °C for 10 s, and 60 °C for 30 s.

### 2.4. H. pylori cagL Sequencing

The primers *cagL*-B4: 5′-GCAGAATTCATAACAAGCGGCTTAAAG-3′ and *cagL*-B5: 5′-ATTAGAATTCATAGCCTATCGTCTCAG-3′ were used for the amplification of 695 bp of fragments during *cagL* sequencing [30]. DreamTaq DNA Polymerase (Thermo Fisher Scientific, Waltham, MA, USA) was used for PCR according to the manufacturer’s recommendations, with an annealing temperature of 55 °C. PCR amplification was verified on a 2% agarose gel and subjected to Sanger sequencing. The *cagL* sequence of *H. pylori* strain P12 (GenBank: ACJ07700.1) was used as a reference. The *cagL* sequences were translated to corresponding amino acid sequences using the sequence alignment program MAFFT [31], and the detection of polymorphisms was performed and visualized using Jalview v2.11.2.4 [32]. Neighbor-joining phylogenetic trees were constructed from both *cagL* nucleotides and translated amino acid sequences using the MEGA11 (version 11.0.13) program with the bootstrap method at 500 replications [30].

### 2.5. Statistical Analysis

Statistical analyses were carried out using IBM SPSS Statistics version 25.0 (IBM Corp., Armonk, NY, USA). The association between CagL amino acid polymorphisms and gastroduodenal diseases was evaluated by Fisher’s exact test. Risk assessments of specific patterns were calculated by binary logistic regression. All statistical tests were two-sided, and a *p* value lower than 0.05 was considered statistically significant.

## 3. Results

In total, 70 out of 99 samples (70.7%) were positive for *H. pylori* DNA in this study. We observed that *H. pylori* DNA positivity was higher in PUD patients, with a percentage of 84.4 (38/46) compared to 60.3 (32/53) in NUD patients.

A positive band for *cagL* was detected in 42 out of 70 (60%) samples (PUD, 23; NUD, 19), and following this, these 42 samples were sequenced. Sequencing data were aligned against the *H. pylori* strain P12 ACJ07700 locus and showed >95% homology. A total of 27 different CagL amino acid polymorphisms and their frequencies were determined and are provided in Table 1 and Supplementary Figure S1. All RGD and FEANE motifs were identical, as the reference sequence showed no variability (Supplementary Figure S1). In the TASLI motif, four polymorphisms were observed: 171T (3/42), 171V (1/42), 172P (1/42), and 174V (1/42). CagLHM showed five different amino acid combinations: NEIGQ (25/42), NKIGQ (11/42), DKIGQ (4/42), NKMGQ (1/42), and DKMGE (1/42) (Table 2). The DKI sequence, known as the East Asia sequence, was observed at a 9.5% frequency.

CagL amino acid polymorphisms 22F and 114M were detected at 100% in both the PUD and NUD patient samples. CagL amino acid polymorphisms and their distributions between groups are given in Table 1. The 84T, 154K, 171V, 172P, and 174V polymorphisms were only detected in PUD patient samples, and the 154Q, 203I, 206S, and 223Q polymorphisms were only detected in NUD patient samples (Table 1). The CagL amino acid polymorphisms among groups showed variability, but there was no statistical significance in any polymorphism alone (*p* > 0.05).

The percentages of CagLHM amino acid polymorphisms and sequences in both the PUD and NUD patient samples are given in Table 1 and Table 2. The NKMGQ and DKMGE sequences were only detected in NUD patient samples. In our study, we did not find any association between the CagLHM sequences and gastroduodenal diseases (*p* > 0.05).

Three CagL amino acid polymorphism combinations associated with PUD and NUD were determined (Table 3). The CagL amino acid polymorphism combinations and their relative risk assessments are given in Table 4. Pattern 1 was only detected in 6 out of 23 PUD patient samples and was associated with a 1.35-fold risk (*p* = 0.02). Patterns 2 and 3 were found in only four NUD patient samples and were associated with a 1.26-fold risk (*p* = 0.03).

**Figure 1:**
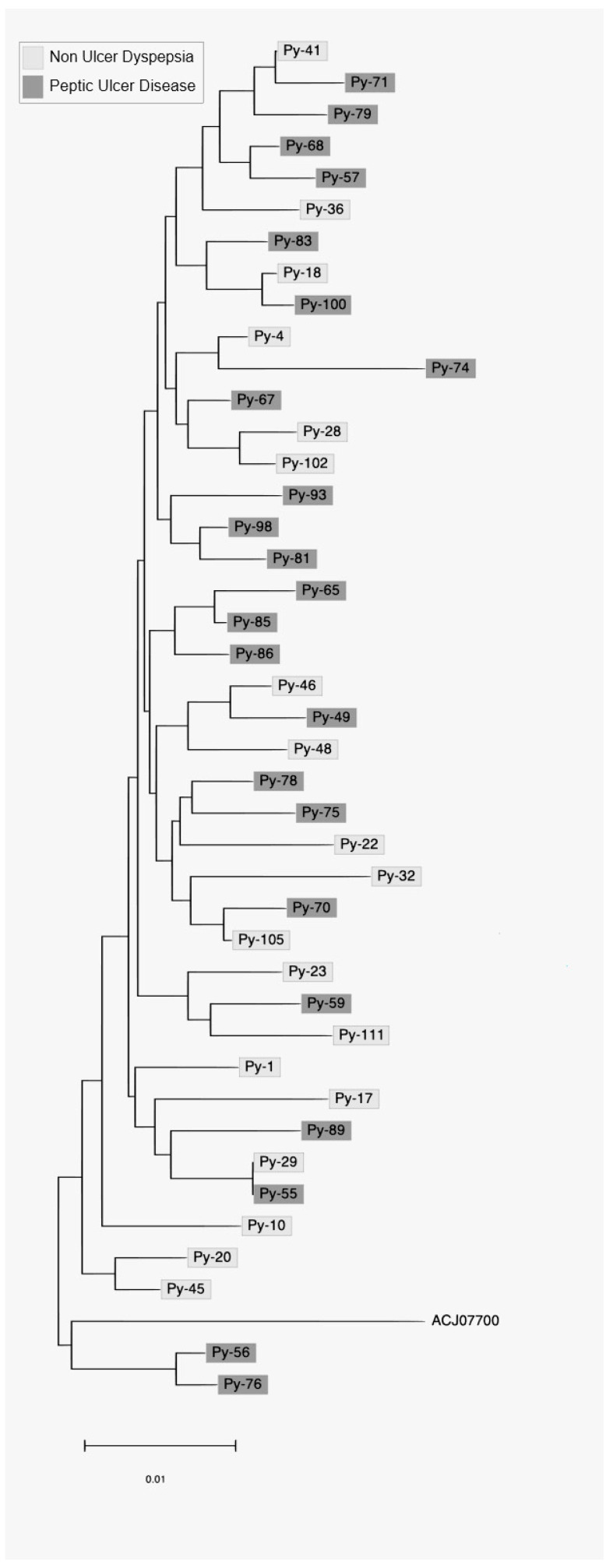
Phylogenetic tree of *H. pylori* strains based on *cagL* nucleotide sequences. No characteristic clusters were observed between PUD and NUD for both the DNA and amino acid sequences of CagL.

The constructed neighbor-joining trees from *cagL* nucleotide and amino acid sequences from 42 samples are presented in Figure 1. No characteristic clusters were observed between PUD and NUD for both the DNA and amino acid sequences of CagL.

## 4. Discussion

The correlation between *H. pylori*-related clinical outcomes and many virulence factors is still an important research topic that will help us understand the pathogenesis of *H. pylori*. Specifically, the EPIYA motif in the *cagA* gene region of *H. pylori* has been shown to be associated with gastric cancer. It has been proven that the EPIYA-D segments in eastern strains of *H. pylori* and the EPIYA-C segments in western strains of *H. pylori* play a role in the development of gastric cancer [2,3]. T4SS plays an important role in the translocation of CagA to the gastric epithelium, which is essential in the gastric carcinogenesis caused by *H. pylori*. The interaction of the CagL protein with integrins in the gastric epithelium has an important role in the binding of *H. pylori* [7,8]. The interaction of CagL with the gastric epithelium and the correlation of CagL amino acid polymorphisms with different clinical outcomes have been investigated in various studies. CagLHM, in particular, is a highly polymorphic region of the CagL protein found between the 58th and 62nd amino acids. It has been reported that *H. pylori* CagL Y58E59 polymorphisms cause an upward shift in integrin α5β1 in the corpus, causing more severe chronic inflammation in the corpus and increasing the risk of gastric cancer [22]. It has also been shown that *H. pylori* CagL-Y58/E59 still maintains its stronger binding affinity to integrin β1, CagA translocation, and IL-8 secretion activity, even under adverse low pH conditions [23]. However, isogenic CagL Y58/E59 variants of *H. pylori* 26695 have been reported to significantly block the translocation and phosphorylation of CagA compared to wild-type CagL [33]. Similarly, it was determined that the transfer of *H. pylori* CagL Y58E59, D58K59, D58E59, N58E59, or N58K59 polymorphisms did not significantly alter CagA translocation and IL-8 secretion [34]. It has been considered that variations at CagL positions 58 and 59 do not affect T4SS function but may instead work in concert with certain polymorphisms elsewhere in CagL to mitigate disease progression [34].

In addition to these, the distribution of the polymorphisms in this region varies among different geographic regions and shows different associations with gastroduodenal diseases. Understanding the association between the geographical differences along with CagL polymorphisms and the clinical outcomes is of great importance, as it can provide very important information about diseases linked to *H. pylori*. Rizzato et al. [20] showed 74 nucleotide polymorphisms in the *cagL* gene in *H. pylori* strains in Mexico and Venezuela and reported that four of them (166, 172, 228, and 516 positions) were associated with gastric cancer. Yeh et al. [22] evaluated residues 58, 59, 122, 201, 210, 216, 221, and 234; and Shukla et al. [21] evaluated residues 35, 58, 59, 60, 62, and 122 in different studies. They showed that polymorphisms in amino acid residues 58 and 59 are associated with gastric cancer risk. Unlike Yeh et al. [22], Shukla et al. [21] reported that D58 and K59 are associated with gastric cancer in India. On the contrary, Cherati et al. [24] reported that the D58 polymorphism is related to PUD but not to gastric cancer in Iran. A study from Mexico stated that D58/K59 polymorphisms are dominant in chronic gastritis patients [26]. A study from Turkey by Ozbey et al. [35] found that D58 polymorphisms are associated with gastric cancer and duodenal ulcers (thesis). Gorrell et al. [27] did not find any association between PUD and CagLHM amino acid polymorphisms in their global analysis. Various correlations of CagLHM amino acid polymorphisms with clinical outcomes in the research show CagL variability in regional *H. pylori* strains. In this study, the CagL protein (sequence between 21 and 237) of *H. pylori* in samples from PUD and NUD patients were analyzed. In total, 27 CagL amino acid polymorphisms were detected. Their distribution among groups shows variability, but there was no statistical significance in any CagL amino acid polymorphism evaluated alone (Table 1). Moreover, we did not find any significant relationship between the CagLHM amino acid polymorphisms and the study groups (Table 1). Although there was no significant difference between groups, the dominance of N58, I60, and Q62 in the PUD group and the 100% presence of I60 and Q62 in the PUD group were striking. Although the reported associations between polymorphisms in the CagLHM region in particular and *H. pylori*-related clinical outcomes have been found in various studies [22,23], the same polymorphism has also been associated with different clinical outcomes [21,24,26], or, similar to our results, no relation has been found [27]. This variability in the association between CagL polymorphisms and clinical outcome suggests that it may be related to factors such as the association of *cagL* with other gene regions or the association of CagL polymorphisms with other CagL polymorphisms. For example, *H. pylori* CagL/or f17 genotypes were found to be risk factors for peptic ulcer [36]. Similarly, multiple EPIYA-C repeats and the CagLHM NEIGQ sequence were reported to be correlated with PUD and gastric cancer risk [30]. Tafreshi et al. [34] also determined that the effect of different polymorphisms at positions 58 and 59 of CagLHM on T4SS function was not different. This suggests that combining various data sets to determine the association of CagL polymorphisms with clinical outcome may be beneficial.

Many researchers have described different amino acid sequences for CagLHM, of which DKMGE, NEIGQ, NKIGQ, and DKIGK are the dominant ones, constituting 75% of all amino acid combinations worldwide. In the Americas, DKMGE, NEIGQ, and -IGK are the most frequent sequences. NEIGQ, NKIGQ, and DKMGE are the most frequent sequences in Europe. The most diverse distributions were observed in Asian countries (a total of 27 different variants), in which NEIGQ and DKIGK were the most prevalent sequences [27]. Yadegar et al. [30] reported 10 different variants, with the most prevalent ones being NEIGQ and NKIGQ, with 45.7% and 19.6% frequencies, respectively, in Iran.

Nevertheless, the worldwide distribution of CagLHM amino acid sequences and their significance in duodenal diseases are important and well-studied. Yadegar et al. [30] found that NKMGK is related to PUD, with a 42.8% prevalence. Roman et al. [26] reported the frequencies of DKMGE (75%), NEIGQ (6.1%), and NKMGQ (6.1%) sequences in chronic gastritis patients. In Thailand, Ogawa et al. [37] reported six different sequences and found that all gastric ulcer and duodenal ulcer patients have the same *H. pylori* with the sequence DKIGK, whereas its frequency in gastritis patients without ulcer is 66.6%. In addition, Ozbey et al. [35] reported a 58-fold risk of gastric cancer in patients infected with the *H. pylori* strain carrying the DKIGQ sequence in our country but did not report a risk for duodenal ulcer (master’s thesis). The distributions of CagLHM amino acid sequences detected in our study were similar in the PUD and NUD groups (Table 2). In our study, we did not find any association between the CagLHM amino acid sequences and gastroduodenal diseases, but their distribution is similar to that of the Asian profile due to the lower percentage of DKMGE, and the other way around due to the lack of diverse sequences.

Other than CagLHM, there are other CagL amino acid polymorphisms related to gastroduodenal diseases. Cherati et al. [24] reported that N122 and K35 are associated with the risk of developing a peptic ulcer, and V134 and N122 increase gastric cancer risk compared to gastritis patients with CagL amino acid polymorphisms I134 and K122. Similarly, the frequencies of amino acid polymorphisms T88, N101, A141, and E142 were found to be increased in gastric cancer patients, and the D201 and V234 amino acid polymorphisms were found to be increased in non-GC patients [37]. Roman et al. [26] reported that K122, I134, M73, and I175 are common polymorphisms among chronic gastritis patients in Mexico.

No significant association between polymorphisms in *cagL* and the clinical outcomes found in our study was detected. However, studies have suggested that it would be beneficial to evaluate all polymorphisms together and not just CagL polymorphisms alone. When we evaluate our data from this point of view, although none of the polymorphisms alone are related to PUD and NUD, their combinations showed associations in our study (Table 4). Pattern 1, which includes the K35, N122, V134, T175, R194, and E210 residues, was found to be associated with a 1.35-fold peptic ulcer risk (*p* = 0.02). The other two patterns were associated with a 1.26-fold NUD risk: pattern 2: V41 and I134 (*p* = 0.03) and pattern 3: V41, K122, A171, and I174 (*p* = 0.03). All three patterns were disease-state specific. Pattern 1 was only observed in the PUD group, and patterns 2 and 3 were only observed in the NUD group. These patterns, which were found to be associated with PUD and NUD in our study, show that the evaluation of the association of polymorphisms at different positions outside of CagLHM may contribute to our understanding of the association of *H. pylori* with clinical outcome. Other CagL polymorphisms, such as the correlation of certain CagLHM polymorphisms with diseases or polymorphisms in other *H. pylori* proteins, are thought to be extremely important to study together in order to gain a better understanding of the association with clinical outcome. The coexistence of polymorphisms in different regions of CagL may possibly affect the overall activity of the protein. Modeling the effect of different polymorphisms on the overall CagL crystal structure may be useful to evaluate how CagL alters structure and function.

There are some limitations to our study. For example, the number of samples in the study groups is small. With a larger sample size, an association can be determined between single amino acid polymorphisms and clinical outcomes. Additionally, with a larger sample size, different patterns associated with different clinical outcomes can be detected. Another limitation of our study is that it did not include a gastric cancer group. Studies involving patients with gastric cancer may contribute to the association of gastric cancer with CagL polymorphisms.

## 5. Conclusions

In conclusion, the diversity of the *H. pylori* CagL amino acid polymorphisms detected in this study matches both European and Asian *H. pylori* strains. We observed three new patterns related to gastroduodenal diseases. Using this combination approach, it can be seen that CagL amino acid polymorphisms other than those in the CagLHM region are important as well. Pattern 1 is related to PUD, and the other two patterns (patterns 2 and 3) are related to NUD. Consideration of these patterns and the evaluation of CagL polymorphisms from this perspective may be useful for understanding the course of *H. pylori* infection. Further investigations on these patterns with a larger sample size that includes gastric cancer patients may be useful in terms of approaches to *H. pylori* infection.

## Figures and Tables

**Table 1:** Distribution of CagL amino acid polymorphisms.

CagL Amino Acid Polymorphisms	PUD ^†^ (n = 23)	NUD ^‡^ (n = 19)	*p* Value
22D/F	23/0	19/0	>0.05
32S/N	6/17	4/15	>0.05
35Q/K	9/14	9/10	>0.05
41V/A/T	18/2/3	17/1/1	>0.05
56A/T	1/22	1/18	>0.05
58D/N	21/2	16/3	>0.05
59K/E	13/10	12/7	>0.05
60M/I	23/0	17/2	>0.05
62E/Q	23/0	18/1	>0.05
84A/T	1/22	0/19	>0.05
114I/M	23/0	19/0	>0.05
122K/N	19/4	15/4	>0.05
134I/V	22/1	15/4	>0.05
154E/Q/K	22/0/1	18/1/0	>0.05
171A/T/V	20/2/1	18/1/0	>0.05
172S/P	1/22	0/19	>0.05
174I/V	1/22	0/19	>0.05
175T/I	1/22	1/18	>0.05
194R/K	2/21	2/17	>0.05
200Q/H	22/1	18/1	>0.05
203V/I	0/23	1/18	>0.05
206N/S	0/23	1/18	>0.05
210E/K	1/22	2/17	>0.05
223R/Q	0/23	1/18	>0.05

^†^ PUD, Peptic Ulcer Disease; ^‡^ NUD, Non-Ulcer Dyspepsia.

**Table 2:** The amino acid sequences detected in CagLHM and their distributions.

CagLHM Amino Acid Sequences	Total n = 42 (%)	PUD ^†^ n = 23 (%)	NUD ^‡^ n = 19 (%)	*p* Value
NEIGQ	25 (59.52)	13 (56.5)	12 (63.1)	>0.05
NKIGQ	11 (26.19)	8 (34.8)	3 (15.8)	>0.05
DKIGQ	4 (9.52)	2 (8.7)	2 (10.5)	>0.05
NKMGQ	1 (2.38)	0 (0)	1 (5.3)	>0.05
DKMGE	1 (2.38)	0 (0)	1 (5.3)	>0.05

^†^ PUD, Peptic Ulcer Disease; ^‡^ NUD, Non-Ulcer Dyspepsia.

**Table 3:** CagL amino acid polymorphism patterns detected in the study.

Patterns	CagL Amino Acid Polymorphism Combinations
Pattern 1	K35/N122/V134/T175/R194/E210
Pattern 2	V41/I134
Pattern 3	V41/K122/A171/I174

**Table 4:** Association of patterns with the risk of PUD and NUD.

Patterns	PUD ^†^ n = 23 (%)	NUD ^‡^ n = 19 (%)	*p* Value	Odds Ratio	95% CI ^‡‡^
1	6 (26.1)	0 (0)	0.02	1.353	1.061–1.725
2	0 (0)	4 (17.4)	0.03	1.26	1.004–1.597
3	0 (0)	4 (17.4)	0.03	1.26	1.004–1.597

^†^ PUD, Peptic Ulcer Disease; ^‡^ NUD, Non-Ulcer Dyspepsia; ^‡‡^ CI, Confidence Interval.

## Data Availability

The data presented in this study are available on reasonable request from the corresponding author.

## Supplementary Materials

The following supporting information can be downloaded at: https://www.mdpi.com/article/10.3390/medicina58121738/s1. Figure S1: Amino acid sequences of CagL obtained from 42 *H. pylori* isolates aligned against *H. pylori* strain P12.
