# Supplementary figures and images for: New CagL Amino Acid Polymorphism Patterns of Helicobacter pylori in Peptic Ulcer and Non-Ulcer Dyspepsia
Source: Medicina (Kaunas). 2022 Nov 27;58(12):1738. doi: 10.3390/medicina58121738 (PMC9782086; doi:10.3390/medicina58121738)

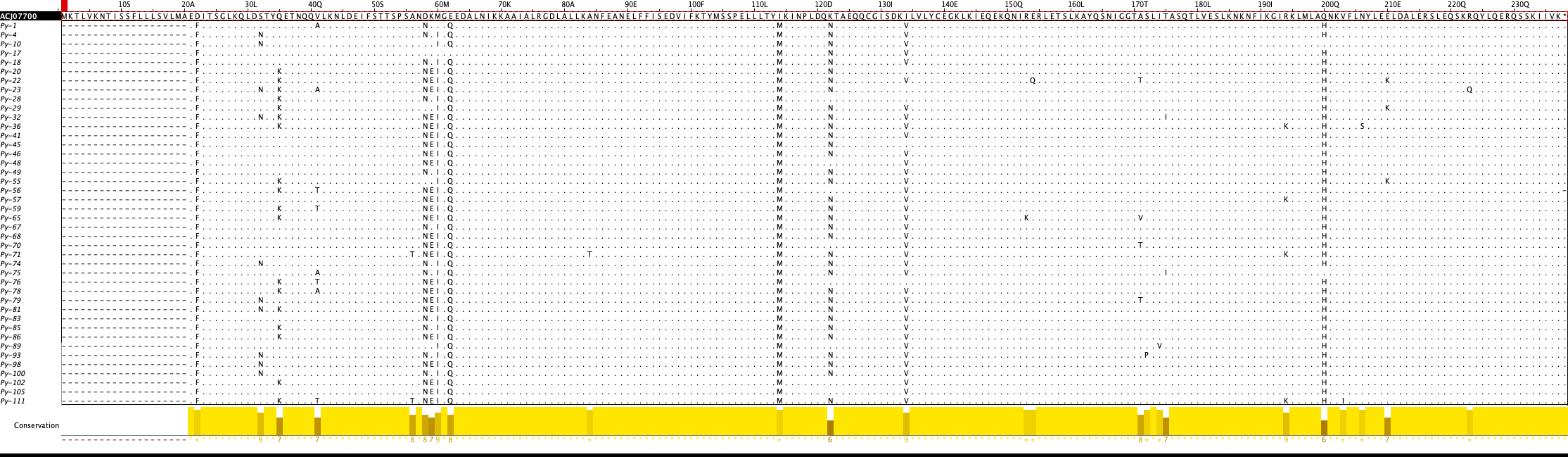

Supplement: Supplementary file 1 [file medicina-58-01738-s001.zip › medicina-1986535-FIgure S1.jpg]
